# Classification models for Tobacco Mosaic Virus and Potato Virus Y using hyperspectral and machine learning techniques

**DOI:** 10.3389/fpls.2023.1211617

**Published:** 2023-10-16

**Authors:** Haitao Chen, Yujing Han, Yongchang Liu, Dongyang Liu, Lianqiang Jiang, Kun Huang, Hongtao Wang, Leifeng Guo, Xinwei Wang, Jie Wang, Wenxin Xue

**Affiliations:** ^1^ Tobacco Research Institute of Chongqing Company, Chongqing, China; ^2^ Tobacco Research Institute, Chinese Academy of Agricultural Sciences, Qingdao, China; ^3^ Science and Technology Department of Sichuan Liangshan Company, Liangshan Yi Autonomous Prefecture, Xichang, China; ^4^ Science and Technology Department of Yunnan Honghe Company, Hani-Yi Autonomous of Honghe Prefecture, Mile, China; ^5^ Agricultural Information Institute, Chinese Academy of Agricultural Sciences, Beijing, China

**Keywords:** precision agriculture, virus diseases, machine learning, hyperspectral, nondestructive

## Abstract

Tobacco Mosaic Virus (TMV) and Potato Virus Y (PVY) pose significant threats to crop production. Non-destructive and accurate surveillance is crucial to effective disease control. In this study, we propose the adoption of hyperspectral and machine learning technologies to discern the type and severity of tobacco leaves affected by PVY and TMV infection. Initially, we applied three preprocessing methods – Multivariate Scattering Correction (MSC), Standard Normal Variate (SNV), and Savitzky-Golay smoothing filter (SavGol) – to corrected the leaf full-length spectral sheet data (350-2500nm). Subsequently, we employed two classifiers, support vector machine (SVM) and random forest (RF), to establish supervised classification models, including binary classification models (healthy/diseased leaves or PVY/TMV infected leaves) and six-class classification models (healthy and various severity levels of diseased leaves). Based on the core evaluation index, our models achieved accuracies in the range of 91–100% in the binary classification. In general, SVM demonstrated superior performance compared to RF in distinguishing leaves infected with PVY and TMV. Different combinations of preprocessing methods and classifiers have distinct capabilities in the six-class classification. Notably, SavGol united with SVM gave an excellent performance in the identification of different PVY severity levels with 98.1% average precision, and also achieved a high recognition rate (96.2%) in the different TMV severity level classifications. The results further highlighted that the effective wavelengths captured by SVM, 700nm and 1800nm, would be valuable for estimating disease severity levels. Our study underscores the efficacy of integrating hyperspectral technology and machine learning, showcasing their potential for accurate and non-destructive monitoring of plant viral diseases.

## 1 Introduction

**Figure 1:**
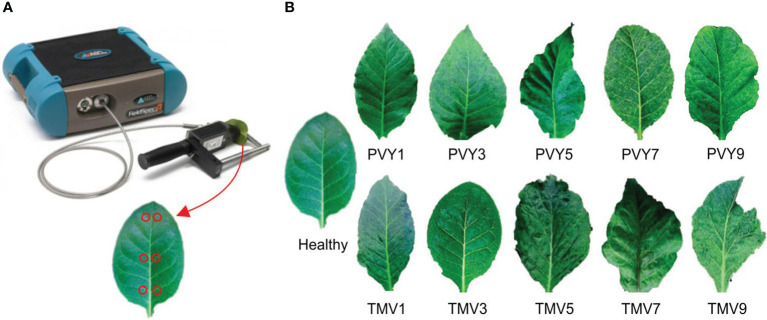
**(A)** The equipment ASD Field Spec4 (up) and the strategy of spectra data collection in this study (down); **(B)** the PVY/TMV diseased leaves with different severity levels (Wang et al., 2020).

Tobacco Mosaic Virus (TMV) and Potato Virus Y (PVY) are widespread virus diseases in fields and cause massive economic losses to crops (Scholthof et al., 2011; Khateri et al., 2014; Korbecka-Glinka et al., 2021). Both PVY and TMV infect a wide range of plants, especially tobacco and other members of the family Solanaceae, causing symptoms such as leaf mosaic, vein clearing, and deformation (McDonald and Singh, 1996; Yang and Klessig, 1996; Quenouille et al., 2013). The key to effective viral disease control is to directly monitor the occurrence and prevalence of diseases. However, the traditional methods of visual or molecular identification are time-consuming, inefficient, and destructive. At the same time, TMV and PVY infections are difficult to be separated and could develop into severe symptoms rapidly under an ideal environment, which leads to missing the best control period (**Figure 1B**). Therefore, automatic identification of the disease occurrence and severity degree of plants in the field in time will be of great benefit for precise prevention by guiding the chemical application where and when needed at an appropriate dose, further controlling the spread of TMV and PVY in time and avoiding great production loss. Furthermore, the automated disease identification can be integrated into innovative disease-resistant plant breeding process, expediting the phenotyping process and yielding time savings compared to the visual assessment by human raters.

Modern agriculture has benefited greatly from the high-tech vision solutions such as artificial intelligence, machine learning, etc. For example, spectrum technology has often been used in precision agriculture to fill gaps in continued human monitoring. Spectral reflectance captures crop biomass, disease information, and crop quality (Gnyp et al., 2014; Martínez-Martínez et al., 2018). The principle of spectroscopic technology is to identify the content and composition of substances using the different characteristics of different substances such as light absorption and reflection (Fang et al., 2015). In particular, at the onset of PVY and TMV infection, leaf structural characteristics and chlorophyll levels begin to change. These changes will further trigger the reflection spectrum. Hence, taking advantage of these fluctuations in the reflection spectrum, we could carry out disease detection and monitoring thanks to modern techniques avoiding irretrievable yield loss caused by missing the best control time. The advantages of applying spectral technology to monitor crop diseases are fast, non-destructive, and wide-area detectable. It has been verified in previous studies that the usage of leaf spectral information could effectively monitor and distinguish leaf disease (Huang et al., 2012; Hu et al., 2016; Kong et al., 2018; Martínez-Martínez et al., 2018; Long et al., 2021; Cai et al., 2022; Fernández et al., 2022). However, there are scarce studies to explore the application value in the detection of virus diseases.

Furthermore, searching for sensitive wavelength bands is the focus of spectroscopic technology applications. The optimal wavelength of the leaf spectrum can be quickly and accurately located by machine learning (Steddom et al., 2005; Li et al., 2022; Lv et al., 2022). Machine learning algorithms can process a large number of data sets with irregular surfaces, find the potential probability distribution of the data, and make predictions, which can be used for the diagnosis and prediction of crop diseases. Machine learning is mainly classified into supervised classification and unsupervised classification. Supervised classification algorithms include support vector machine (SVM), random forest (RF), decision Tree, KNN algorithm, linear regression, and other methods often used in the binary classification regression analysis. Unsupervised classification algorithms are mainly used for cluster analysis. Most of the applications have been implemented using supervised variants of machine learning algorithms rather than unsupervised ones. Besides, among the above algorithms, RF training speed is fast, having strong model generalization ability. The SVM algorithm is suitable for analyzing finite samples, overcoming the shortcomings of some other binary support vector machines, and improving multi-classification accuracy (Uddin et al., 2019).

Machine learning holds immense potential for enhancing accuracy. Hence, the fusion of spectral data and machine learning techniques has been harnessed for leaf disease diagnosis (Lamba et al., 2021). Nevertheless, spectral data often carries inherent noise, making the careful selection of appropriate algorithms and models paramount in the accurate identification of tobacco diseases. By analyzing the full-length spectra (350-2500nm) of healthy and diseased tobacco leaves, this study aims to establish robust and effective classification models for TMV and PVY diseases by two classifiers: support vector machine (SVM) and random forest (RF) (**Figure 1**). The outcomes of this research will be helpful to facilitate the early-stage disease type and the severity level assessments, scientifically informed strategies for preventing and controlling leaf diseases.

## 2 Materials and methods

### 2.1 Test materials and the test equipment

The study was performed in the JiMo experimental area of the Tobacco Research Institute, Chinese Academy of Agricultural Sciences, Qingdao City, China (120.58°N, 36.45°E). The TMV or PVY^N^ pathogens and tobacco seedlings K326 were provided by the Plant Protection Institute of the Chinese Academy of Agricultural Sciences.

The test equipment is ASD Field Spec4 portable handheld ground object spectrometer, which is equipped with VNIR (350-1000nm) 512-pixel silicon array detector in the visible region, SWIR1 (1001-1800nm) graded index InGaAs detector, and SWIR2 (1801-2500mm) graded index InGaAs detector. The acquisition wavelength range is 350-2500nm, the wavelength reproducibility is 0.1nm, and the wavelength accuracy is 0.5nm.

### 2.2 Test methods

#### 2.2.1 Disease inoculation

The tobacco plant variety is K326 cultivated to the 7-8 leaf stage under the greenhouse conditions (25 ± 1°C, 65% ± 5% relative humidity, and 14:10h light: dark photoperiod). After the leaves were dusted, the healthy leaves were inoculated with pathogens TMV or PVY by mechanical inoculation method according to (Piche et al. (2004).

#### 2.2.2 Spectral data acquisition

Parameters of the FieldSpec4 equipment were adjusted according to the usage specifications (**Figure 1A**). The optical fiber probe was at a 5° angle of view, and a distance of 10 cm above the blade surface for measurement. The lens was aimed at the whiteboard to optimize the instrument, and then the lens was moved to the tested leaf to store the leaf reflectance spectrum data. The whiteboard optimization was done for every ten tobacco plants measured.

Tobacco plants at 7-8 leaf stages were selected for measurement. Six measurement points were picked on each tobacco leaf at the base of the leaf, the middle of the leaf, and the top of the leaf using the leaf vein as the axis of symmetry (**Figure 1A**). The average value of these six points was taken as the spectral reflectance of the leaf.

#### 2.2.3 Disease data collection

Six disease severity grades were applied to this study according to the disease grading standard GB/T23222-2008, namely, healthy leaf (no symptoms on the whole plant), grade 1 (Zero to one-quarter of the leaf is mosaic), grade 3 (one quarter to one-third of the leaf is mosaic), grade 5 (one third to a half of the leaf is mosaic, slight deformation or slightly darken vein), grade 7 (a half to two-thirds of the leaf is mosaic, deformation or vein necrosis), and grade 9 (two thirds to the whole leaf is mosaic, severe deformation or severe vein necrosis) (**Figure 1B**).

The spectral reflectance of healthy and TMV or PVY diseased leaves at the five unhealthy classes of grade 1 (TMV1 or PVY1), grade 3 (TMV3 or PVY3), grade 5 (TMV5 or PVY5), grade 7 (TMV7 or PVY7) and grade 9 (TMV9 or PVY9) were collected respectively, using the same method in 2.2.2. 893 samples were obtained in total (**Table 1**). A number of 286 samples were obtained for TMV-diseased leaves, 456 for PVY-diseased leaves, and 151 for healthy leaves (**Table 1**).

**Table 1:** Spectrum data collection of tobacco diseased leaf and healthy leaf.

	training sample	test sample	sample size
healthy	125	26	151
PVY	361	95	456
TMV	229	57	286
PVY1	20	4	24
PVY3	24	10	34
PVY5	96	22	118
PVY7	120	34	154
PVY9	102	24	126
TMV1	37	14	51
TMV3	60	10	70
TMV5	47	12	59
TMV7	36	13	49
TMV9	45	11	56
Diseased	589	153	742

### 2.3 Data processing

#### 2.3.1 Data preprocessing

The collected spectral data includes over 2150 wavenumber points. In the actual spectral data acquisition process, the environmental conditions, sampling time, sampling points, and so on would affect the collecting result by inducing scattering and noises. Therefore, before the model was built, the original spectral data were perpetrated by three data preprocessing methods: Multivariate Scattering Correction (MSC), Standard Normal Variate (SNV), and Savitzky-Golay smoothing filter (SavGol).

MSC is one of the common methods of hyperspectral data preprocessing, which can effectively eliminate the spectral difference caused by the scattering level and correct the baseline shift, and offset the phenomenon of spectral data (Windig et al., 2008). The formulas are as follows:

a. The average of all spectral data as the “ideal spectrum”


(1) $$\begin{array}{c} \vec{Data}=\frac{\sum_{i=1}^{n}Data_{ij}}{n} \end{array}$$

b. The baseline translation and offset of each sample were obtained by solving the least squares problem by unary linear regression between the spectrum of each sample and the average spectrum.


(2) $$\begin{array}{c} Data_{i}=k_{i}\vec{Data}+b_{i} \end{array}$$

c. The spectrum of each sample was corrected by subtracting the obtained baseline translation and dividing by the offset.


(3) $$\begin{array}{c} Data_{i\left(MSC\right)}=\frac{\left(Data_{i}-b_{i}\right)}{k_{i}} \end{array}$$

SNV was used to eliminate the effects of solid particle size, surface scattering, and optical path variation on the near-infrared band (NIR) diffuse reflectance spectra (Dhanoa et al., 1995). The formula is:


(4) $$\begin{array}{c} X_{i,SNV=\frac{x_{i,k}-\bar{x_{i}}}{\sqrt{\frac{\sum_{k=1}^{m}\left(x_{k}-\bar{x_{i}}\right)}{\left(m-1\right)}}}}\; \end{array}$$

*x_i_* is the average of the spectra of the i sample; k=1,2,…, m. m is the wave point; i=1,2,…,n;

n is the corrected sample number; *X_i, SNV_* is the transformed spectrum.

SavGol could improve the smoothness of the spectrum, reduce the interference of noise, and ensure that the shape and width of the signal remain unchanged while filtering out the noise (Savitzky and Golay, 1964; Mishra et al., 2019). SavGol smoothing uses polynomial functions to smooth signals. It involves selecting a window, fitting a polynomial to the data within it, and replacing the central point with the polynomial’s value. The window size and polynomial choice are typically manual, based on visual inspection. In our work, we used a second-order polynomial and a 5-point window for smoothing.

#### 2.3.2 Model establishment

Since spectral data may have multicollinearity, SVM and RF algorithms are used to prevent over-fitting. All the data set was divided into two parts: 80% as the training set, and the remaining 20% of the data set as the test set to evaluate the performance of the trained algorithms in the test set.

The SVM algorithm divides the optimal hyperplane by constructing feature space. The idea of maximizing the classification margin is the core of the SVM method. SVM contains several parameters, such as kernel function, gamma value, and penalty factor C (Boser et al., 1992; Cortes and Vapnik, 1995). In this study, the Linear is used as the kernel function. The optimal model parameters were determined by GridSearch in the training set based on 50% fold cross-validation. The penalty factor Cs were 1 for SVM + MSC, 40 for SVM + SNV, 1 for SVM + SavGol.

RF algorithm is an algorithm, integrated with a large number of decision trees. The final predicted result is obtained according to the summary of the scores of the decision tree nodes on the dataset. Each decision tree classifies the input vector, and the final classification result is determined by the vote of each tree. Therefore, the number of decision trees is the most important parameter affecting RF, and the optimal number of decision trees is determined by GridSearch based on 50% fold cross-validation in the training set (Breiman, 2001; Liaw and Wiener, 2002). The numbers of decision trees were 53 for RF + MSC, 39 for RF + SNV and 25 for RF + SavGol.

**Figure 2:**
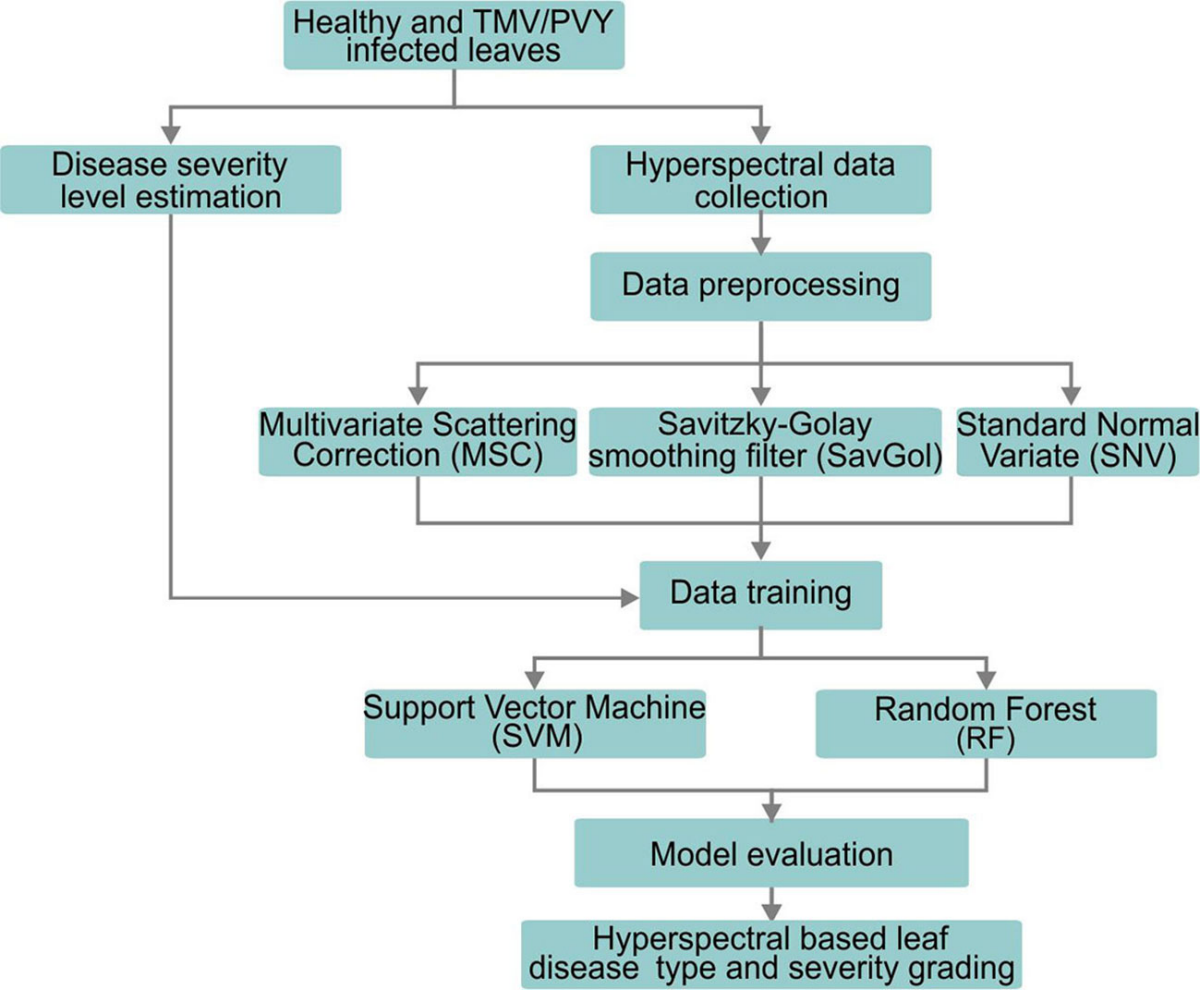
Workflow of this study.

The binary classification is the identification of healthy and diseased tobacco leaves, or PVY diseased leaves and TMV diseased leaves. The six-class classification is suffered TMV grades TMV1, TMV3, TMV5, TMV7, TMV9 and healthy leaves, respectively, and suffered PVY grades PVY1, PVY3, PVY5, PVY7, PVY9 and healthy leaves. The sample dataset was randomly divided into a training set and a test set according to 8:2 (Details information was listed in **Table 1**). Each algorithm was trained with the best parameters through the training set, and the trained algorithm evaluated the performance of the model on the test set (**Figure 2**).

#### 2.3.3 Model performance evaluation

Evaluation indicators of each classifier: “Precision” means the ratio of the number of correctly classified samples of a certain category to the predicted samples of this category; “Recall” means the ratio of the number of correctly classified samples of a certain category to the real number of the category, f1 is the comprehensive evaluation of “precision” and “recall”; “accuracy” means the proportion of the number of samples that are correctly predicted; “support” means the number of samples (Lamba et al., 2021).


(5) $$\begin{array}{c} precision=\frac{\left(TP\right)}{\left(TP+FP\right)} \end{array}$$

(6) $$\begin{array}{c} Recall=\frac{TP}{\left(TP+FN\right)} \end{array}$$

(7) $$\begin{array}{c} F1_{score}=\frac{\left(2*TP\right)}{\left(2*TP+FN+FP\right)} \end{array}$$

(8) $$\begin{array}{c} Accuracy=\frac{\left(TP+TN\right)}{\left(TP+TN+FP+FN\right)} \end{array}$$

TP is the number of true positive; TN is the number of true negative; FP is the number of false positive; FN is the number of negative.

As more attention is paid to diagnostic accuracy in agricultural disease diagnosis, precision scores are the main evaluation index in this study. The higher the precision score, the better performance of the model will be.

### 2.4 Data analysis

There are three different methods of data preprocessing and two classifiers to create six combinations of algorithmic models (**Table 2**). All data processing was run by Python3.9 and R 4.0.1 versions.

**Table 2:** The average precision (%) of each algorithm combination in different classification models.

algorithm models	Binary classification		Six-class classification	
	healthy and diseased leaf	PVY and TMV	TMV	PVY
SavGol+SVM	97.4	100.0	96.2	98.1
MSC+SVM	97.0	99.5	96.2	97.4
SNV+SVM	97.4	98.0	96.2	96.9
SavGol+RF	97.4	100.0	98.4	88.7
MSC+RF	96.6	90.3	88.7	76.6
SNV+RF	97.0	97.0	85.2	91.5

## 3 Results

### 3.1 Spectral data preprocessing

**Figure 3:**
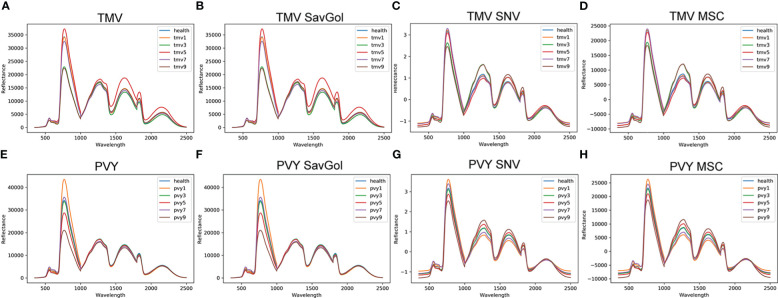
The leaf reflectance of tobacco leaves. **(A)** PVY: spectra without any correction; **(B)** PVY MSC corrected spectra; **(C)** PVY: SavGol corrected spectra; **(D)** PVY: SNV corrected spectra; **(E)** TMV: spectra without any correction; **(F)** TMV: MSC corrected spectra; **(G)** TMV: SavGol corrected spectra; **(H)** TMV: SNV corrected spectra.

Mean spectral reflectance curves under different conditions are shown in **Figure 3**. We could see that the spectral reflectance patterns of various kinds of leaves give the same variation trend (**Figures 3A, E**). In general, there are three significant peaks around 780nm, 1250nm, and 1600nm and one valley value under the near-infrared band in healthy, PVY, or TMV-infected leaves. Under the above three peaks, there are different degrees of overlaps among the healthy and various levels of diseased leaves.

After different pretreatments, the spectra’ reflectance changed in different ways. For TMV-infected leaves, SNV (**Figure 3C**) and MSC (**Figure 3D**) modified the spectral reflectance significantly in the near-infrared band (NIR), while producing overlaps of spectral reflectance around the 780nm band. Similar to a scenario in the TMV experiment, pretreatments of SNV (**Figure 3G**) and MSC (**Figure 3H**) increased the reflectance discrimination ability of different severity levels of PVY diseased leaves but reduced the spectral resolution ability around the 780nm band.

Overall, MSC and SNV preprocessing methods revealed a relatively outstanding ability to improve the NIR band’s spectral resolution. The spectral reflectance after SavGol treatment did not change significantly. In addition, the resolution of PVY-diseased leaves is better than TMV-diseased leaves (**Figure 3**).

### 3.2 Tobacco leaf disease binary classification

#### 3.2.1 The binary classification of healthy leaf and diseased leaf

**Figure 4:**
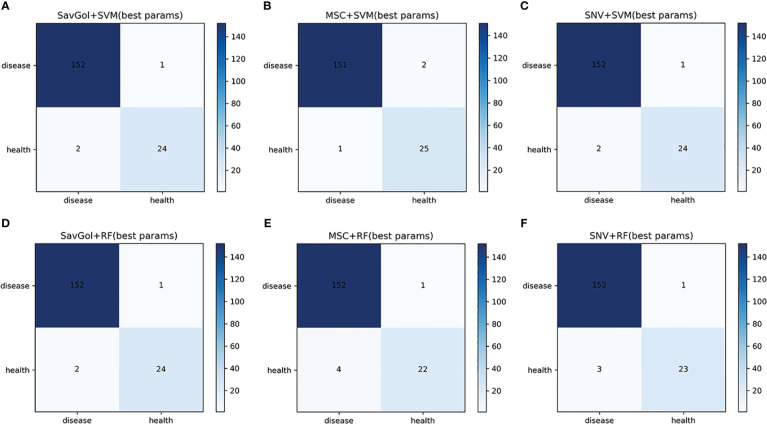
The confusion matrix of each algorithm model in the binary classification of healthy leaves and diseased leaves. **A–F**: Number of correctly and misclassified samples for six classification models: **(A)** SavGol+SVM, **(B)** MSC+SVM, **(C)** SNV+ SVM, **(D)** SavGol+RF, **(E)** MSC+RF, and **(F)** SNV+RF.

The number of 893 samples was randomly divided into a training set (80% with 714 samples) and a test set (20% with 179 samples) (**Table 1**). As shown in **Tables 2**, **3**; **Figure 4**, in the binary classification of healthy leaf and diseased leaf, all the preprocessing methods and classifiers gave high recognition precision and accuracy of healthy leaves with over 93%. The misclassification for the RF classifier mainly came from the mistaken healthy leaves of diseased ones (**Figure 4**). The recognition precision of SavGol+RF, SavGol+SVM, and SNV+SVM combinations reached up to 98% (**Table 2**), which could be potentially adopted for the accurate identification of diseased leaves and healthy leaves.

**Table 3:** Evaluation index scores of each algorithm model in the binary classification of healthy leaf and diseased leaf.

algorithm models	leaf	precision	recall	f1 - score	support	accuracy
SavGol+SVM	diseased	0.987	0.993	0.99	153	0.983
	healthy	0.96	0.923	0.941	26	
SavGol+RF	diseased	0.987	0.993	0.99	153	0.983
	healthy	0.96	0.923	0.941	26	
MSC+SVM	diseased	0.987	0.987	0.99	153	0.983
	healthy	0.926	0.962	0.943	26	
MSC+RF	diseased	0.974	0.993	0.984	153	0.972
	healthy	0.957	0.846	0.898	26	
SNV+SVM	diseased	0.987	0.993	0.99	153	0.983
	healthy	0.96	0.923	0.941	26	
SNV+RF	diseased	0.981	0.993	0.987	153	0.978
	healthy	0.958	0.885	0.92	26	

#### 3.2.2 The binary classification of PVY diseased leaf and TMV diseased leaf

**Figure 5:**
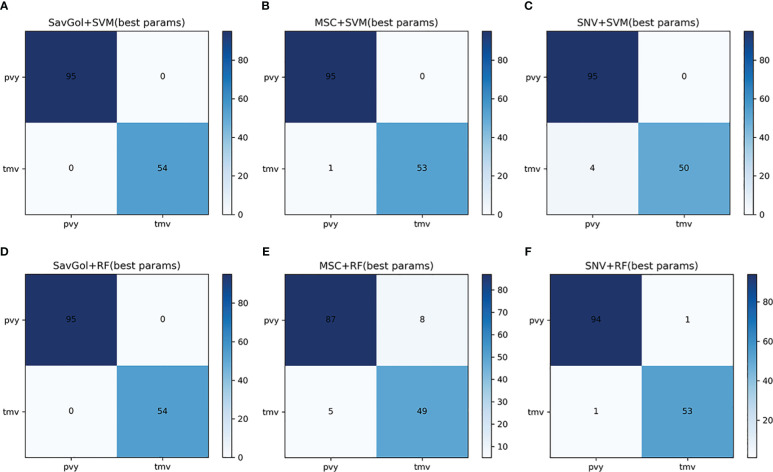
The confusion matrix of each algorithm model in the binary classification of PVY diseased leaf and TMV diseased leaf. **A–F**: Number of correctly and misclassified samples for six classification models: **(A)** SavGol+SVM, **(B)** MSC+SVM, **(C)** SNV+ SVM, **(D)** SavGol+RF, **(E)** MSC+RF, and **(F)** SNV+RF, respectively.

A total of 742 samples were input for the classification of PVY diseased leaf and TMV diseased leaf. 590 samples for the training set and 152 samples for the test set. **Tables 2**, **4**; **Figure 5** showed that in the identification of PVY diseased leaves, the average precision after the SavGol pretreatment method was the highest at 100%. The recognition precision of TMV-diseased leaves by the combination of MSC+RF was lower than 86% (**Table 4**), and most of the errors were misjudging TMV-diseased leaves as PVY diseased leaves (**Figure 5**). The overall classification result was better when combined SVM classifier. The algorithm models of SavGol+RF, SavGol+SVM, and MSC+SVM combinations could greatly help achieve the accurate identification of TMV and PVY diseases.

**Table 4:** Evaluation index score of each algorithm model in the binary classification of PVY-infected leaf and TMV-infected leaf.

algorithm models	disease	precision	recall	f1-score	support	accuracy
SavGol+SVM	PVY	1.00	1.00	1.00	95	1.00
	TMV	1.00	1.00	1.00	54	
SavGol+RF	PVY	1.00	1.00	1.00	95	1.00
	TMV	1.00	1.00	1.00	54	
MSC+SVM	PVY	0.99	1.00	0.995	95	0.993
	TMV	1.00	0.981	0.991	54	
MSC+RF	PVY	0.946	0.916	0.935	95	0.913
	TMV	0.86	0.907	0.891	54	
SNV+SVM	PVY	0.959	1.00	0.979	95	0.973
	TMV	1.00	0.926	0.962	54	
SNV+RF	PVY	0.989	0.989	0.989	95	0.986
	TMV	0.981	0.981	0.981	54	

### 3.3 TMV six-class classification

**Figure 6:**
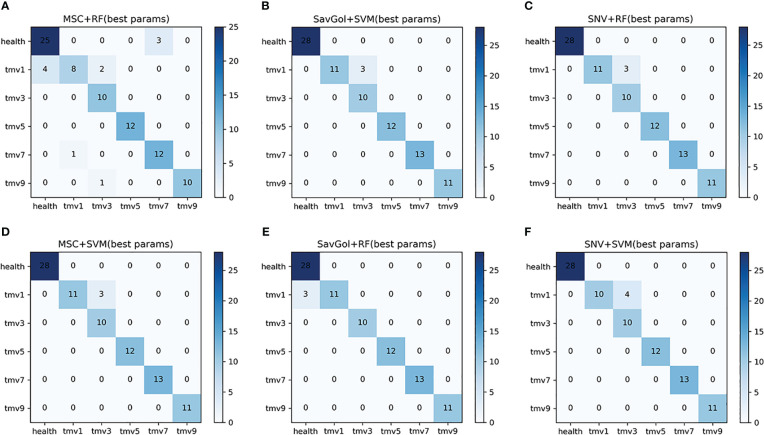
The confusion matrix of each algorithm model in the six-class classification of TMV diseased leaf. **(A–F)**: Number of correctly and misclassified samples for six classification models: **(A)** MSC+RF, **(B)** SavGol+SVM, **(C)** SNV+RF, **(D)** MSC+SVM, **(E)** SavGol+RF, and **(F)** SNV+ SVM.

There are 437 samples for the TMV six-class classification. 349 samples were randomly separated into the training set, and 88 samples in the test set (**Table 1**). In the TMV six-class classification, it is known from **Table 5**; **Figure 6** that in the cases of healthy, TMV1, TMV5, TMV7, and TMV9 recognition, most of the models have performed excellently. Unlike the mediocre performance of PVY3 identification, the recognition precisions of the TMV3 leaf were relatively poor in all models (**Table 5**).

**Table 5:** Evaluation index score of the six-class classification of TMV diseased leaf.

algorithm models	severity grade	precision	recall	f1-score	support	accuracy
SavGol+SVM	healthy	1.00	1.00	1.00	28	0.966
	TMV1	1.00	0.786	0.88	14	
	TMV3	0.769	1.00	0.869	10	
	TMV5	1.00	1.00	1.00	12	
	TMV7	1.00	1.00	1.00	13	
	TMV9	1.00	1.00	1.00	11	
SavGol+RF	healthy	0.903	1.00	0.949	28	0.966
	TMV1	1.00	0.786	0.88	14	
	TMV3	1.00	1.00	1.00	10	
	TMV5	1.00	1.00	1.00	12	
	TMV7	1.00	1.00	1.00	13	
	TMV9	1.00	1.00	1.00	11	
MSC+SVM	healthy	1.00	1.00	1.00	28	0.966
	TMV1	1.00	0.786	0.88	14	
	TMV3	0.769	1.00	0.869	10	
	TMV5	1.00	1.00	1.00	12	
	TMV7	1.00	1.00	1.00	13	
	TMV9	1.00	1.00	1.00	11	
MSC+RF	Healthy	0.862	0.893	0.877	28	0.875
	TMV1	0.889	0.571	0.696	14	
	TMV3	0.769	1.00	0.869	10	
	TMV5	1.00	1.00	1.00	12	
	TMV7	0.80	0.923	0.857	13	
	TMV9	1.00	0.909	0.952	11	
SNV+RF	healthy	1.00	1.00	1.00	28	0.966
	TMV1	1.00	0.786	0.88	14	
	TMV3	0.769	1.00	0.869	10	
	TMV5	1.00	1.00	1.00	12	
	TMV7	1.00	1.00	1.00	13	
	TMV9	1.00	1.00	1.00	11	
SNV+SVM	healthy	1.00	1.00	1.00	28	0.955
	TMV1	1.00	0.714	0.833	14	
	TMV3	0.714	1.00	0.833	10	
	TMV5	1.00	1.00	1.00	12	
	TMV7	1.00	1.00	1.00	13	
	TMV9	1.00	1.00	1.00	11	

We could see that both classifiers had better recognition of healthy leaves and TMV5, and the misjudgments were mostly concentrated in TMV1 and TMV3. For example, the errors of models using SVM as a classifier were mostly misjudgments of TMV1 to TMV3. The misjudgments of combinations including RF as a classifier were mainly at the TMV1 level (**Figure 6**). The classification precision of SavGol+RF for different TMV disease grades was the highest, with a rate of 98% (**Table 2**).

**Figure 7:**
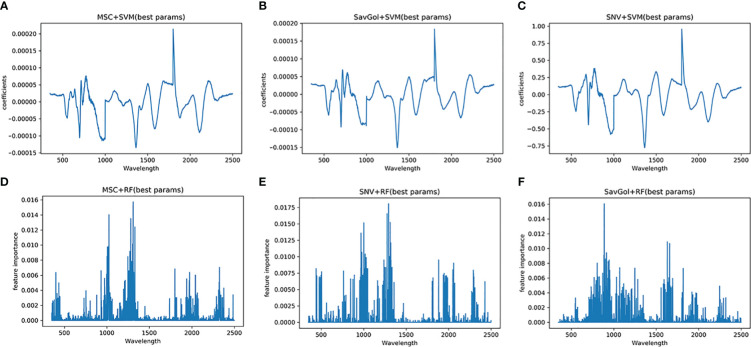
Characteristic wavelength maps of TMV diseased leaf. The spectral signatures of each wavelength were calculated by two algorithms (SVM and RF) with three pretreatment methods (MSC, SNV and SavGol): **(A)** MSC+SVM, **(B)** SavGol+SVM, **(C)** SNV+ SVM, **(D)** MSC+RF, **(E)** SNV+RF, and **(F)** SavGol+RF. Height of peaks indicate the contribution of each wavelength to the predictive power of the model.

The full spectral analysis revealed that all bands contributed fluctuated information. **Figure 7** shows the effective bands captured by the combination of MSC+SVM, SavGol+SVM, and SNV+SVM were concentrated around 1801nm and 1802nm. The effective bands captured by the combinations with the RF classifier dispersed extremely. These may indicate that the different important bands captured by different classifiers may be one of the important reasons that affect the recognition accuracy.

### 3.4 PVY six-class classification

A total of 607 samples was collected for PVY six-class classification analysis, among which 485 samples were randomly divided into the training set and 122 samples were into the test set.

In the results of the PVY six-class classification, the performance of the same preprocessing method and classifier varied greatly among the severity grade classifications. In the recognition of severity levels PVY1 and PVY3, the precisions of models including the SVM classifier were 100% while RF has a poor estimation ability with low precision rates. For the recognition of PVY5, only the models after SavGol pretreated came up to 90%. For PVY7 and PVY9 identification, all models are generally excellent with high precision rates between 96% and 100% (**Table 6**).

**Table 6:** Evaluation index score of each algorithm model in the PVY diseased leaf six-class classification.

algorithm models	severity grade	precision	recall	f1-score	support	accuracy
SavGol+SVM	healthy	0.966	1.00	0.982	28	0.975
	PVY1	1.00	1.00	1.00	4	
	PVY3	1.00	0.90	0.947	10	
	PVY5	0.917	1.00	0.957	22	
	PVY7	1.00	0.94	0.97	34	
	PVY9	1.00	1.00	1.00	24	
SavGol+RF	healthy	0.844	0.96	0.90	28	0.918
	PVY1	0.667	1.00	0.80	4	
	PVY3	1.00	0.70	0.824	10	
	PVY5	0.909	0.91	0.909	22	
	PVY7	1.00	0.88	0.938	34	
	PVY9	0.96	1.00	0.98	24	
MSC+SVM	healthy	0.964	0.964	0.964	28	0.967
	PVY1	1.00	1.00	1.00	4	
	PVY3	1.00	0.90	0.947	10	
	PVY5	0.88	1.00	0.936	22	
	PVY7	1.00	0.941	0.97	34	
	PVY9	1.00	1.00	1.00	24	
MSC+RF	healthy	0.565	0.929	0.703	28	0.762
	PVY1	0.571	1.00	0.727	4	
	PVY3	0.80	0.40	0.533	10	
	PVY5	0.692	0.409	0.529	22	
	PVY7	0.968	0.882	0.923	34	
	PVY9	1.00	0.833	0.909	24	
SNV+SVM	healthy	0.966	1.00	0.982	28	0.959
	PVY1	1.00	1.00	1.00	4	
	PVY3	1.00	0.70	0.824	10	
	PVY5	0.846	1.00	0.917	22	
	PVY7	1.00	0.94	0.97	34	
	PVY9	1.00	1.00	1.00	24	
SNV+RF	healthy	0.90	0.68	0.931	28	0.943
	PVY1	0.80	1.00	0.889	4	
	PVY3	0.875	0.70	0.778	10	
	PVY5	0.917	1.00	0.957	22	
	PVY7	1.00	0.91	0.954	34	
	PVY9	1.00	1.00	1.00	24	

**Figure 8:**
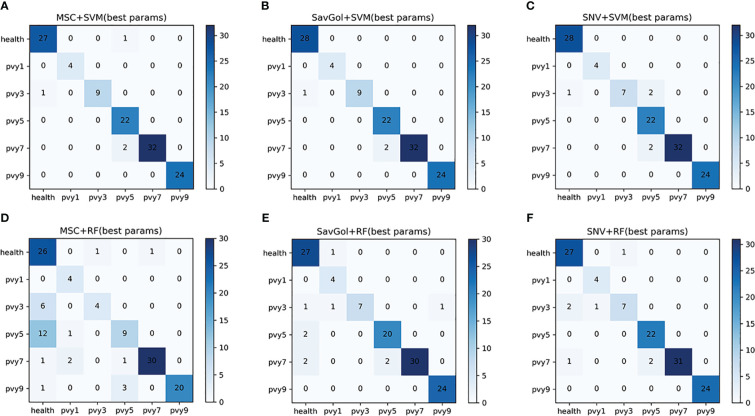
The confusion matrix of each algorithm model in the six-class classification of PVY diseased leaf. **(A–F)**: Number of correctly and misclassified samples for six classification models: **(A)** MSC+SVM, **(B)** SavGol+SVM, **(C)** SNV+ SVM, **(D)** MSC+RF, **(E)** SavGol+RF, and **(F)** SNV+RF.

Overall, the results showed that the precision rates of the models combined with the SVM classifier were high (**Table 6**). The errors centered on misidentifying PVY3 as healthy leaves. For the RF classifier, mainly misjudged PVY3 and PVY5 (**Figure 8B**). The SavGol+SVM was awarded the best model among six combinations in our data set, with an average precision of 98%, which could be used to identify different disease grades of PVY (**Table 2**).

**Figure 9:**
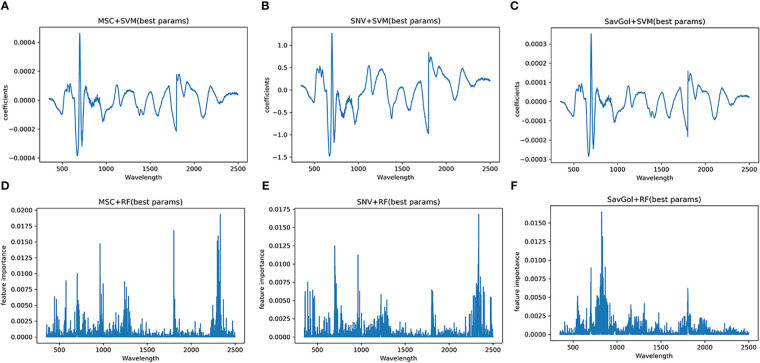
Characteristic wavelength maps of PVY diseased leaf. The spectral signatures of each wavelength were calculated by two algorithms (SVM and RF) with three pretreatment methods (MSC, SNV and SavGol): **(A)** MSC+SVM, **(B)** SNV+ SVM, **(C)** SavGol+SVM, **(D)** MSC+RF, **(E)** SNV+RF, and **(F)** SavGol+RF. Height of peaks indicate the contribution of each wavelength to the predictive power of the model.

The pattern of captured characteristic bands is similar to the TMV experiment. The effective bands of PVY-diseased leaves captured by different treatment combinations were different (**Figure 9**). Three combinations showed better recognition performances, SavGol +SVM, MSC+SVM, and SNV+SVM. The feature bands contributing more information to the model building were relatively centralized, 699nm, 698nm, 700nm, and other near-infrared bands. However, the important bands caught by combinations using RF as a classifier are highly dispersed. For SNV+RF, they are far infrared bands such as 2338nm and 962nm, while those captured by SavGol +RF are near-infrared bands such as 826nm, 835nm, and 867nm. For MSC+RF, they are far infrared bands around 2331nm, 1803nm, and 2307nm.

## 4 Discussion

Spectroscopy has been used in precision agriculture for fast and non-destructive determining crop disease epidemic situations (Martínez-Martínez et al., 2018; San-Blas et al., 2020; Cai et al., 2022; Fernández et al., 2022; Li et al., 2022; Lv et al., 2022). Machine learning in leaf disease recognition is superior to traditional methods such as regression and clustering analyses (Ingale and Baru, 2019). Combined with automatic extracting spectral characteristics to improve the precision of the model, machine learning could be better applied to precision agriculture to guide the diagnosis of agricultural diseases. Here, these two technologies (hyperspectral and machine learning) were utilized to diagnose the type and severity degree of virus diseases PVY and TMV on tobacco. The optimal model for plant virus disease diagnosis was explored by setting up six combinations with three spectral data preprocessing methods (MSC, SNC, and SavGol) and two machine learning classifiers (SVM and RF).

Overall, all the models have excellent capabilities in the identification of the type and infection severities of TMV/PVY diseased leaves. In the binary classification model, the SVM classifier performed better compared to RF with over 97% precision (**Table 2**). Using an SVM classifier, Fernández et al. (2022) also achieved a good separation effect of yellow powder disease four days after cucumber leaves inoculation, and the overall accuracy rate was above 95% (Fernández et al., 2022). For the six-class classification, the performance of the SVM classifier is also obviously good and the best combination is SavGol+SVM (**Table 2**). However, our results illustrate that different classifiers have distinct diagnostic precisions for different diseases, which is similar to the diagnosis result of rice diseases using SVM (Sethy et al., 2020). The errors of different classifiers were not completely consistent either (**Figures 6**, **8**), which might be caused by the slight deviations from the original manual definition of severity grades.

Lastly, machine learning was also used to automatically extract spectral features (Barradas et al., 2021). The results illustrated the effective wavelengths for identifying diseases located in visible and near-infrared light bands. In detail, the characteristic wavelengths for identifying PVY diseases were concentrated in the vicinity of 700nm, which was similar to the sensitive wavelength of other leaf diseases (Long et al., 2021). A previous study discovered a strong correlation between the reflectance of the band near 700nm and chlorophyll content, carotene, and other leaf pigments (Chen et al., 2012). Besides, when the leaf is infected by disease pathogens, the visible area will have higher reflectance (Shi et al., 2009; Sankaran and Ehsani, 2013; Wang et al., 2014). The characteristic wavelength of TMV-diseased leaves captured by SVM is near 1800nm. The near-infrared band is related to the internal structure and dry matter of leaves. Therefore, the change in the internal structure of leaves infected by the virus will change the spectral reflectance. But most studies were focused on the spectral wavelength range of 380–1023nm, which only found a similar effective wavelength of around 700nm (Xie et al., 2015; Zhu et al., 2017; Sethy et al., 2020). Here, the full-length spectra study revealed the potential candidate effective bands in the NIR region. Based on the captured effective bands, it is clear that even the spectral reflectance resolution became higher after MSC and SNV treatment in the NIR, but the identification abilities of the models with MSC or SNV were still lower than models with SavGol.

In yet other words, different spectral features could be caused by different diseases, while various feature bands might be caught by diverse classifiers for the same disease further leading to irregular classification accuracies (Kong et al., 2018). Therefore, all the combinations using SVM as a classifier captured the same effective bands of the same disease, while the combinations including the RF classifier had a huge variation in the effective wavelength capture for the same disease leaves. This may be one reason that the RF classifier has underperformed in this scenario.

## 5 Conclusions

Given the precise plant disease management, early detection plays a pivotal role in guiding timely interventions and preventing potential losses in production. Identification of the disease type proves invaluable in selecting the proper control strategies and expediting the breeding process. Moreover, the rapid development speed of tobacco viral diseases in the field underscores the challenge of sustaining minimal impact over time. Thus, assessing infection severities becomes crucial in choosing the appropriate intensity of control efforts. This study facilitates a comprehensive investigation into a rapid and non-invasion diagnostic model of the type and severity grades of two important virus disease -TMV and PVY, and further explore the optimal classification models. These classifiers process the categorization of tobacco-diseased leaf severity by capturing the feature wavelengths, paving the way for future large-scale promotion and application. Take, for instance, unmanned aerial vehicle (UAV)-based hyperspectral platforms, which have gained significant prominence due to their lightweight, flexibility, and ease of operation for detecting plant diseases (Vanegas et al., 2018). Nevertheless, there are still certain limitations to consider. Specifically, the acquisition cost of hyperspectral data remains relatively high, regardless of financial or labor expenses. External factors can also influence image quality during data collection, including factors such as measurement timing, light intensity, solar altitude angle, and more.

To sum up, all the classification models examined in this study demonstrated commendable performance in distinguishing PVY and TMV diseases in tobacco. Moreover, the SVM classifier did a better job than RF in the binary and six-class classification of PVY and TMV-diseased tobacco leaves. Additionally, the synergy between the SavGol preprocessing method and the SVM classifier yielded exceptional precision rates exceeding 96% across all classification tasks. In the light of feature wavelengths caught by SVM, specifically the 700nm band of PVY diseased leaf and the 1800nm band of TMV diseased leaf holds significant promise for the development of PVY and TMV disease classification model in the future large-scale monitoring, such as UAV spectral detection. In short, the integration of hyperspectral technology and machine learning offers a promising avenue for early detection of PVY and TMV disease leaves to achieve effective crop management.

## Data availability statement

The original contributions presented in the study are included in the article/supplementary material. Further inquiries can be directed to the corresponding authors.

## Author contributions

XW, HC, YH, JW, and WX designed the experiments. HC, YH, YL, DL, LJ, KH, and HW performed the experiments. HC, YH, XW, JW, and WX analyzed the data. YH and HC wrote the manuscript with input from LG, XW, JW, and WX. All authors contributed to the article and approved the submitted version.
